# Organisational opportunities for person-centred care in residential aged care: a focus group study with managers and staff

**DOI:** 10.1136/bmjopen-2026-118924

**Published:** 2026-07-31

**Authors:** Roar Hermansen Østby, Synneve Dahlin-Ivanoff, Jenna Najar, Qarin Lood

**Affiliations:** 1 Department of Health and Rehabilitation, Institute of Neuroscience and Physiology, Sahlgrenska Academy, University of Gothenburg, Gothenburg, Sweden; 2 Centre for Person-Centred Care, University of Gothenburg, Gothenburg, Sweden; 3 Department of Psychiatry and Neurochemistry, Institute of Neuroscience and Physiology, Sahlgrenska Academy, University of Gothenburg, Gothenburg, Sweden; 4 Alzheimer Center Amsterdam, Neurology, Vrije Universiteit Amsterdam, Amsterdam UMC Location VUmc, Amsterdam, Amsterdam, The Netherlands; 5 Amsterdam Neuroscience, Neurodegeneration, Amsterdam, The Netherlands

**Keywords:** Person-Centered Care, Patient-Centered Care, Organisation of health services, Nursing Homes

## Abstract

**Abstract:**

**Objectives:**

To explore organisational opportunities for person-centred care in residential aged care facilities from the perspectives of managers and staff.

**Design:**

A qualitative study using focus group discussions and inductive thematic analysis followed by a deductive interpretation guided by a theoretical framework.

**Setting:**

Residential aged care facilities providing long-term care for older people in Sweden.

**Participants:**

A total of 23 participants were recruited using purposive sampling and participated in five focus groups. Participants included assistant nurses, occupational therapists, physiotherapists, registered nurses, social service managers and healthcare managers working in residential aged care. To be included, participants had to have experience of working in residential aged care.

**Results:**

The analysis generated two interconnected themes. The inductively developed theme, *Creating foundations for person-centred care*, described organisational conditions perceived as necessary for enabling person-centred care in residential aged care. Three subthemes highlighted the importance of establishing a safe and supportive environment, maintaining continuity of care and ensuring responsiveness to older persons’ desires and needs. The deductively developed theme, *Turning foundations for person-centred care into action,* interpreted these organisational foundations in relation to the three routines of the University of Gothenburg Centre for Person-Centred Care framework: initiating, working and safeguarding the partnership. Partnership was understood as a way in which organisational foundations could be enacted in everyday care, rather than as a separate inductive finding.

**Conclusions:**

Person-centred care in residential aged care was described to require organisational conditions that support safe and supportive environments, continuity of care and responsiveness to older persons’ preferences and needs. The findings suggest that workforce stability, managerial support, clear responsibilities, communication and active use of documentation are important for enabling person-centred care in everyday practice. Future initiatives should focus on developing and evaluating organisational approaches that support person-centred care in collaboration with older persons, relatives and staff.

**STRENGTHS AND LIMITATIONS OF THIS STUDY**

- Participants from various professional groups in residential aged care facilities, providing diverse perspectives on organisational conditions for person-centred care.
- Focus group discussions enabled interaction, collective reflection and exploration of shared experiences related to everyday care and leadership practice.
- The combination of inductive thematic analysis and deductive interpretation strengthened the analytical depth, but the deductive approach may also have influenced how the findings were interpreted.
- Perspectives were limited to managers and staff; older persons living in residential aged care facilities and relatives were not included, which restricts the understanding of organisational conditions from their perspectives.
- Recruitment of staff was mediated through managers, which may have influenced who was invited or agreed to participate.

## Background

 In response to population ageing and the increasing prevalence of chronic conditions, healthcare and social service systems worldwide are shifting towards people-centred integrated care.^1^ A central element of this approach is interprofessional and interorganisational collaboration with a person-centred approach, with the aim of effectively integrating internal organisational resources.^2^ Person-centred care seeks to combine professional expertise with recognition of all human beings as capable persons who are interconnected and dependent on one another.^3^ It emphasises shared decision-making and acknowledges the dynamic balance between independence, dependence and interdependence among both those in need of care and those providing it.^4 5^ However, research in residential aged care facilities has identified persistent challenges to person-centred care, including tokenistic involvement and limited opportunities for older persons to influence daily activities and care decisions.^6–8^

In Sweden, admission to a residential aged care facility requires people to be aged 65 years or older and to have undergone a professional assessment indicating a need for care and support that cannot be met in ordinary housing.^9 10^ Persons living in these facilities are therefore typically frail and may experience varying degrees of cognitive impairment, ranging from mild decline to severe symptoms. This makes this group particularly vulnerable to fragmented healthcare and social services. Such fragmentation is characterised by insufficient information transfer and unclear responsibilities for treatment and care.^11 12^ It has been associated with depersonalised care, economic inefficiency,^11^ frequent and potentially avoidable hospital admissions, inappropriate medication use and increased morbidity and mortality.^13–15^ In contrast, person-centred care has been shown to improve health outcomes and quality of life among older persons.^16–18^

Internationally, person-centred care is mandated within residential aged care through legislation, regulations and quality standards.^19–21^ In addition, a European standard specifies minimum requirements for person-centred care.^22^ This standard provides healthcare and social service organisations with structured and transparent guidance for implementation and evaluation.^22^ Nevertheless, challenges remain in applying person-centred care in residential aged care facilities. Our previous research with older persons has identified a need for a cultural shift towards more person-centred care in residential aged care.^8 23^ Contemporary residential aged care has been shown to be influenced by institutional cultures,^23^ referring to the shared norms, values and practices within an organisation.^24^ These cultures also include unspoken rules that shape behaviour, thinking and adaptation.^25^ Such cultures may limit opportunities for staff to involve older persons in meaningful activities and decision-making. They may also constrain the use and development of professional skills and competence.^23 26^ Addressing these challenges requires a re-conceptualisation of institutional cultures towards a more person-centred orientation.^27^

As emphasised by Britten *et al*,^4^ effective and sustainable implementation of person-centred care necessitates coordinated efforts at both practical and organisational levels.^4^ Understanding factors that facilitate or hinder the enactment of person-centred care among managers and staff is therefore essential.^27–31^

Complementing previous research on person-centred care in residential aged care facilities, this study forms part of a larger research project exploring person-centred approaches to residential aged care from multiple perspectives. These include the perspectives of older persons living in residential aged care facilities,^23^ healthcare and social service staff^7^ and managers. While these perspectives are all essential for understanding how person-centred care is experienced and enacted in practice, less is known about the organisational conditions that enable or constrain person-centred care in residential aged care. In particular, there is a need to better understand how such conditions are perceived by those responsible for managing and providing healthcare and social services. Addressing this knowledge gap, the aim of this study was to explore organisational opportunities for person-centred care in residential aged care from the perspective of managers and staff.

## Methods

### Design

Focus group methodology was applied to give participants the opportunity to discuss different aspects of their experiences.^32 33^ Originating from a social constructivist foundation, focus group methodology puts emphasis on the interaction between group participants, unlike many other methods within the qualitative paradigm.^34^ This approach is particularly suitable in studies exploring organisational culture and interprofessional practices, as the group interaction helps illuminate shared norms, collective experiences and tacit understandings that may not emerge in individual interviews.^34^ The report of the study follows the COnsolidated criteria for REporting Qualitative research (COREQ),^35^ see online supplemental material.

### Study setting

The study was conducted in residential aged care in Sweden. Assistant nurses carers, and registered nurses are typically available round-the-clock, whereas registered professionals such as occupational therapists and physiotherapists are working by demand. Managers are generally responsible for developing and implementing organisational guidelines and to set the agenda for the healthcare and social services that are provided in the facility.

### Participants

To generate meaningful and relevant data through dynamic and informative discussions, methodological literature indicates the need for purposive sampling to achieve both homogeneity and heterogeneity within and between groups.^32 34^ Homogeneity was sought by recruiting participants with experience of working in residential aged care and by organising focus groups according to professional field. Heterogeneity was sought by including participants from different residential aged care facilities located in both urban and rural areas, of different sexes and with varying lengths of work experience.

Recruitment was conducted in several steps. Initial contact with the participating facilities was facilitated through a contact person within municipal healthcare and social services. The contact person disseminated information about the study to managers responsible for residential aged care and forwarded expressions of interest to the research team. The contact person did not act as a gatekeeper or selection agent. In total, social service managers from seven facilities expressed interest and were invited to a digital information meeting with the research team. Following this meeting, managers from three facilities contacted the first author by email to confirm their interest in participating. These managers were then asked to assist by identifying eligible staff members and providing their contact details to the research team. Eligible participants were staff with experience of working in residential aged care within healthcare or social services. Healthcare managers and staff were contacted by the last author in her role as a clinical lecturer in municipal healthcare and social services. All contacted staff members agreed to participate. Social service and healthcare managers were also invited to participate because they represented a relevant group due to their responsibility for healthcare and social services in residential aged care. Two out of a total of six healthcare managers declined participation due to lack of time.

All eligible persons received written and verbal information about the aim of the study, what participation would involve and that participation was voluntary. Persons who accepted the invitation were contacted again by telephone to arrange the date, time and place for the focus group discussion. Written informed consent was obtained from all participants before the focus groups were conducted.

In total, 28 persons from three residential aged care facilities were invited to participate, of whom 23 consented and participated in the study. The participants were divided into five focus groups, each consisting of three to seven participants. Each group participated on one occasion. The final sample included 20 women and 3 men, employed as managers (n=7), assistant nurses (n=6), occupational therapists (n=4), physiotherapists (n=3) and registered nurses (n=3). Participants in each focus group were familiar with one another, as they worked closely in teams at the residential aged care facilities. Participants’ ages ranged from 24 to 68 years, and their work experience in residential aged care ranged from 1 to 35 years. For details on participant characteristics, see table 1. All data in the table has been rounded to the nearest integer.

**Table 1:** Participant characteristics

	Male	Female	Age range (years)	Work experience range (years)
Focus group 1: social service managers, n=3	1	2	34–39	1–7
Focus group 2: assistant nurses n=6	2	4	24–55	2–35
Focus group 3: occupational therapists, n=4 and physiotherapists, n=3	0	7	33–68	3–28
Focus group 4: registered nurses, n=3	0	3	46–63	6–27
Focus group 5: healthcare managers, n=4	0	4	44–61	1–12

### Procedure

The focus groups were conducted between May and October 2024, were moderated by the last (group 1) and first (group 2–5) authors and lasted between 39 and 62 min. Four focus groups were conducted at the participants’ workplaces, and one was conducted online. They all started with the moderator presenting the aim and conduct of the study, complementing written information that the participants had received before the focus group. All participants were informed verbally as well as in writing and signed an informed consent form before the discussions were initiated.

The discussions revolved around four key questions developed specifically for this study to study its objective. For clarity, person-centred care was operationalised into involvement of frail older persons in healthcare and social services, resulting in the following key questions:

1. What opportunities do you have for involving frail older persons in healthcare and social services?
2. What barriers do you experience with regard to involving frail older persons in healthcare and social services?
3. Which are the success factors for involving frail older persons in healthcare and social services?
4. Which changes do you wish to see within the organisation?

Participants were encouraged to discuss these questions openly with each other and the moderator sometimes asked follow-up questions such as ‘Can you please elaborate on that?’ or ‘Do you have an example?’ to facilitate a dynamic discussion. The moderator was also attentive to make sure that all persons had the opportunity to express themselves. All focus groups were audio-recorded and professionally transcribed verbatim for analysis. Field notes were also taken during the discussions for contextual information on tone, group dynamics and body language. Transcripts and findings were not returned to participants for comments or corrections since focus group data is socially constructed within the group context.

### Data analysis

Data were analysed using Krueger and Casey’s^32^ stepwise approach to focus group analysis. The analysis proceeded in several stages. First, all audio recordings were listened to repeatedly by the first and second authors to gain an overall understanding of the discussions and their social context. Second, the first author read the transcripts and field notes several times to become thoroughly familiar with the data. During this stage, preliminary interpretations were written down in a separate word document to formulate potential themes with condensed data that described the themes’ meaning. These potential themes were discussed iteratively among all authors and refined through comparison and consensus into a final thematic structure. Following the inductive analysis, the codes, extracts and inductively developed findings were analysed using the evidence-based^4^ University of Gothenburg Centre for Person-Centred Care (GPCC) framework.^3^ This framework comprises three routines for person-centred care: initiating the partnership, working the partnership and safeguarding the partnership.^3^ Thus, text was sorted into the three routines and analysed to formulate deductive themes that outline strategies for operationalising person-centred care in residential aged care. This two-stage analytical process allowed organisational opportunities for person-centred care to be identified inductively from participants’ accounts and subsequently interpreted in relation to an established theoretical framework. Data were managed using Microsoft Word. The analysis did not involve the construction of a formal coding tree; instead, coding and categorisation were conducted iteratively and inductively. The analysis was conducted in Swedish to preserve meaning and nuance. Translation into English was undertaken only after the final thematic structure had been agreed on. All names used in the results section are fictitious.

### Patient and public involvement

A reference group with representatives for pensioneer organisations in Sweden was involved in the design of the study. The group provided input on the relevance and clarity of the study aim and the planned data generation procedures. Their involvement helped ensure that the study addressed issues considered important from the perspective of older persons.

## Results

**Figure 1:**
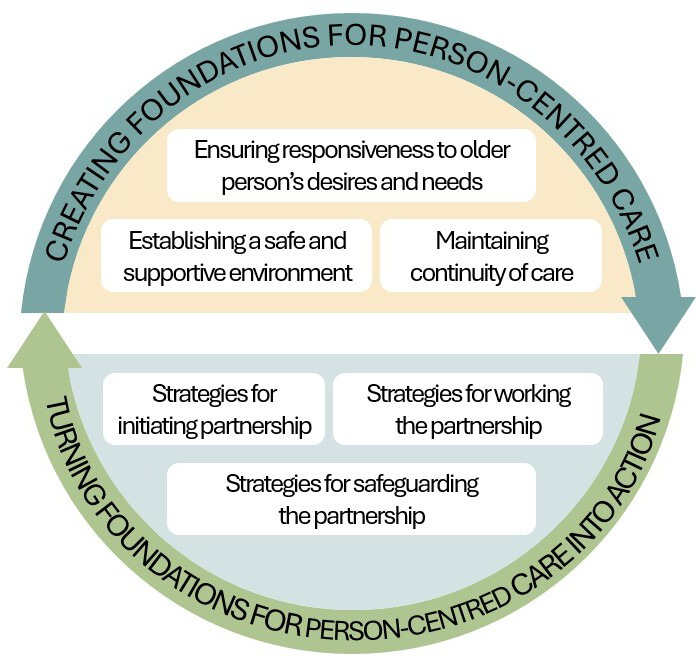
Visualisation of the thematic structure.

The analysis resulted in a two-level thematic structure. The first level was developed inductively and generated the theme *Creating foundations for person-centred care*. This theme captures the organisational conditions and practices that managers and staff perceived as necessary for enabling person-centred care in residential aged care facilities. The second level was developed deductively and generated the theme *Turning foundations for person-centred care into action*. This theme shows how the organisational foundations identified in the inductive analysis could be interpreted in relation to the three routines for person-centred care described in the GPCC framework^3^: initiating, working and safeguarding the partnership. The two analytical levels are connected but serve different purposes. While the inductive theme describes what participants perceived as organisationally important for person-centred care to become possible, the deductive analysis describes how these organisational foundations may support person-centred care in everyday practice. Thus, the findings move from identifying organisational prerequisites to exploring how these prerequisites can be turned into action. The thematic structure is visualised in figure 1.

### Creating foundations for person-centred care

This inductively developed theme captures organisational conditions perceived as necessary for enabling person-centred care in residential aged care. Participants expressed a clear aspiration to work in a person-centred way. However, this aspiration was shaped by organisational conditions such as staff continuity, time, managerial support, professional roles, documentation routines and regulatory requirements. These conditions did not merely affect isolated care activities. Rather, they influenced whether relationships between staff and older persons could develop, and whether older persons’ preferences, needs and life situations could be recognised in everyday care.

Participants described person-centred care as requiring both willingness and determination among managers and staff to identify, seize and, when necessary, actively create opportunities for older persons’ involvement in care decisions and everyday activities. When staff had time, support and continuity, they were better able to interpret older persons’ preferences, involve them in decisions and adapt care and activities to individual needs and wishes. When these conditions were lacking, care risked becoming fragmented, task-oriented and less responsive to the older person. The theme is illustrated by the following quotation from focus group one with social service managers, which highlights the importance of clear structures, defined responsibilities and relational leadership.

Emanuel: Implementation plan, yes exactly. Then risk assessments, as it can be.

Louise: And action plans.

Emanuel: Yes, action plans, exactly. Then we have all the representative roles too. Like, someone is a rehab representative, someone is a documentation representative… And we noticed that we wanted to go that way because if you hadn’t signed that this is my responsibility, then maybe you didn’t really take responsibility…

Lily: Then we also work a lot with relationship building. Being present managers. Being on site as much as possible. And if you aren’t, then you are always at least available by phone, SMS, email. Yes, we are out a lot in the unit and department and have morning coffee and build some personal contacts too. Then it’s easier to come to us if there’s something.

The theme is elaborated through three subthemes that describe key organisational opportunities for person-centred care in residential aged care: *Establishing a safe and supportive environment, Maintaining continuity of care* and *Ensuring responsiveness to older persons’ desires and needs*.

#### Establishing a safe and supportive environment

This subtheme concerns the organisational structures, procedures and managerial support that enabled staff to prioritise the safety and well-being of older persons while recognising their life stories, preferences and social networks. Participants emphasised that staff needed sufficient time and organisational support to understand older persons’ needs and wishes. This involved balancing individual preferences with functional abilities, facility regulations and everyday routines.

Managers were described as central to establishing a safe and supportive environment. They clarified roles and responsibilities, supported prioritisation and helped prevent professional work from being displaced by administrative uncertainty, or organisational disputes. In this way, managerial support protected staff’s ability to focus on older persons’ needs. When staff felt supported, they could be present in the moment, adapt their working methods to individual situations and act in ways that promoted person-centred care. The following quotation from focus group five with healthcare managers concerns uncertainty about responsibilities for basic equipment and assistive devices. It illustrates how managerial support helped clarify roles and ensure that staff could focus on meeting older persons’ needs.

Anna-May: So that we can clarify to the facility managers [social service managers] if they might have an incorrect image or wrong expectations of what our employee should do. Then we can take that route too. So that the facility manager understands what the registered staff, what our mission is.

Ella: We have also worked a lot with prioritising, that is, which patients we should take and how we should prioritise and that part. Or have worked, we are working with, I should say, present tense. Constantly ongoing improvement work.

Ava: And I have, for example, a facility where rehab has said that we need basic equipment, you [as a manager] need to buy this and that and that it is the facility’s responsibility… This thing with assistive devices is gigantic [for us as managers]. It is about renting and buying, so they [social service managers and staff] can get all the help they need, which they are very uncertain about. Because no matter where you work as a unit manager, there are very many different things we do, I think.

Ella: And it is an important role we have too, I think, to be able to ensure that our employees can perform what they are supposed to do, that is, their main task and not end up in discussions about basic equipment or not, but that they can, that the patients get what they need. So that they don’t end up in other administrative, or other power struggles, or anything else.

#### Maintaining continuity of care

This subtheme highlights organisational opportunities for continuity of care, particularly relational continuity. Participants described continuity as dependent on stable staffing, coordinated care processes, documented care plans and repeated interaction between older persons and staff. Relational continuity was difficult to achieve when staff turnover was high, when temporary staff lacked knowledge of older persons’ preferences or when information was not transferred effectively between staff members. Continuity influenced person-centred care by enabling staff to know older persons over time. This familiarity made it easier to recognise subtle changes in behaviour, mood, communication or health, and to understand how different older persons expressed discomfort, preferences or resistance. In contrast, limited continuity weakened the conditions for trust, interpretation and responsiveness.

Action plans were described as important tools for supporting continuity because they documented older persons’ wishes and helped create a shared understanding of roles and responsibilities. Regular meetings involving staff, older persons and relatives were also viewed as important, particularly for persons living with dementia. These meetings created opportunities to update knowledge about the person and to align care with changing needs. The importance of relational continuity is illustrated by the following quotation from focus group four with registered nurses.

Charlotte: It relies a lot on having built some relationships, that you know them. So you know how they react. And know when, like today, no, now we need to step back.

Olivia: … It is important for them that you are on time for them, that you listen to them, what they say. Some take time… even if they don’t have a diagnosis, dementia, some have impaired function. It takes time to talk to them. I have a patient, a blind one, two. They can only see a little, you can write or stand in front and talk, they look at the lips, when you talk to him, he understands what you are saying, or you write on paper. They are all different. Some don’t know the language, they need someone [who knows their language].

#### Ensuring responsiveness to older persons’ desires and needs

This subtheme reflects the importance of attentiveness and flexibility in everyday care. Responsiveness involved having sufficient time to listen and attend to what older persons communicated, both verbally and non-verbally. Participants emphasised the importance of observing body language, facial expressions and bodily movements to identify unspoken needs, especially for persons living with dementia who did not share a common language with staff. Responsiveness was described as both a relational and organisational practice. On the relational level, it was described to require competence, sensitivity and time to interpret individual expressions, as illustrated by a quotation from focus group two with assistant nurses:

Sarah: It is important [to have competence and experience]. If you don’t know the person and haven’t worked in care, you can notice it sometimes when working with new staff. You might think “Oh, does she think I am rushing and doing this too quickly?” But I know that if I don’t do it this way, this person will become upset, because they do not want to be disturbed and made anxious.

Mark: Exactly. That shows how important these life stories are, which we used to work with much more before. Then you had a [documented] life story, perhaps even from the persons themselves, but also from others. You could read a little about how they had lived. And then you can try things. If it said that the person used to eat yoghurt, perhaps I put out yoghurt, and then they say, “No, take they yoghurt away.” But at least you have a chance to try.

At the organisational level, responsiveness required routines that allowed flexibility. Staff needed practical possibilities to adapt routines, return later if the timing was not right, involve relatives, use interpreters or accompany older persons on outings when such wishes were expressed. Communication barriers related to dementia or language differences often required additional time and effort. When organisational conditions did not allow such flexibility, older persons’ preferences risked being overlooked. Conversely, when staff were given time and flexibility, responsiveness became a way of realising person-centred care in everyday encounters.

### Turning foundations for person-centred care into action

This deductively derived theme describes how the organisational foundations identified in the inductive analysis could be translated into concrete actions for person-centred care. Interpreted through the GPCC framework,^3^ the theme comprises three strategy areas corresponding to the routines for person-centred care, described in the subthemes: *Strategies for initiating the partnership, Strategies for working the partnership* and *Strategies for safeguarding the partnership*.

The strategy areas should not be understood as separate empirical findings independent of the inductive analysis. Rather, they represent a deductive interpretation of how the organisational foundations may enable or constrain person-centred care in everyday practice. *Establishing a safe and supportive environment* created conditions for initiating the partnership by supporting trust, openness and dialogue. *Maintaining continuity of care* created conditions for working the partnership over time by sustaining relationships and shared understanding. *Ensuring responsiveness to older persons’ desires and needs* created conditions for safeguarding the partnership by ensuring that older persons’ preferences, needs and agreed action plans were recognised, documented, followed up and acted on in daily care.

#### Strategies for initiating the partnership

Initiating the partnership involved listening to and acknowledging older persons’ narratives, particularly during transitions into residential aged care. Participants described this as a gradual process rather than a single conversation. It required time, trust and communicative flexibility so that older persons and relatives could share experiences, preferences, concerns and expectations. Life stories were described as important for understanding the person, although participants noted that this way of working did not always occur systematically, as illustrated by the following quote from focus group two with assistant nurses.

Lisa: Exactly. And people change. For example, if you have a stroke, you may become a completely different person in your thoughts, so to speak. So these life stories are actually very important. We used to work much more with them before.

Moderator: Has that decreased?

Lisa: Yes, life stories do not come naturally at all now.

Mark: It feels a bit outdated. It feels as though they need to be made more modern somehow, but I do not know how*.*

Strategies for initiating the partnership focused on inclusivity, active listening and effective communication among staff, older persons and relatives. Such strategies were described to enable staff to approach the older person as a person with experiences, preferences and relationships, rather than a recipient of predefined care routines and included:

- Creating a safe environment in which older persons and relatives feel comfortable sharing their ideas and concerns, for example, by encouraging open dialogue and providing supportive spaces for communication.
- Allocating time for attentive listening and relationship building to get to know the older persons and their relatives on a personal level, understand their preferences and build trust over time.
- Paying close attention to non-verbal communication to understand unspoken needs and emotions, particularly among older persons with communication difficulties.
- Adapting communication methods through visual aids, simple language, observation, and collaboration with relatives.

#### Strategies for working the partnership

Working the partnership centred on the realisation of shared decision-making among older persons, relatives, staff, and managers. This required continuity, communication, competence development and managerial support. Participants described the need to translate older persons’ preferences into everyday decisions, both in relation to health-related issues and daily life. This was particularly important when older persons had cognitive impairment, communication difficulties or fluctuating needs. The importance of adapting communication and returning to the person when the timing was more suitable is illustrated by the following quotation from focus group three with occupational therapists and physiotherapists.

Mila: Talk to the person, ask the person. Check body language if they cannot speak. How they act, react when I make a certain movement or lift an arm or turn someone. You notice, is the person scared? Yes, but it is clearly visible. Then they flap or grab or something like that, or grimace if they are in pain or get scared or something. So you communicate with the whole body. It’s just about being attentive to that communication method.

Aurora: And give [them] a lot of time. And maybe come back several times if it wasn’t the right day or whatever it might be. Come back another time.

Beth: I think that when you, I mean we work with each other quite a lot and with several different people, so I would say that overall, I think, of course you should be critical, you can always get better, but I think many of my closes colleagues are very good at it overall. Being attentive, talking to the person. Even if they can’t always, maybe perceive everything exactly.

Strategies for working the partnership focused on continuity, communication, competence development and managerial support. The strategies included:

- Supporting continuity through regular meetings and collaborative action planning, providing a platform for all involved persons to share their perspectives.
- Attending closely to older persons’ reactions and responses, particularly among those with cognitive impairments.
- Using annual surveys as a complement to ongoing dialogue with older persons and relatives.
- Investing in continuous competence development (e.g., regular training sessions, professional development opportunities and language training) to enhance skills and reduce staff turnover.
- Providing managerial support that balances oversight with trust in staff’s professional judgement, enabling adaptation of working methods to meet the unique needs of each older person.

#### Strategies for safeguarding the partnership

Safeguarding the partnership focused on ensuring that older persons’ wishes, needs and agreed action plans were documented, communicated and acted upon in daily care. This routine depended on organisational systems that preserved knowledge about the person despite staff changes, professional boundaries and changing care needs. Documentation, action plans, clearly assigned responsibilities and regular follow-ups were therefore central.

The importance of documenting personal preferences, including seemingly small everyday wishes, is illustrated by the following quotation from focus group two with assistant nurses.

Mark: What you might wish is that they a little earlier, that people are a bit smart, that they write a letter how they want things when they are 75. A little more. But then of course, it is difficult to know how you want things, if I get a stroke, how do I want things then?

Lisa: So, if it were now sort of?

Mark: Yes, something like that. I think that if you imagine yourself at 95, perhaps bedridden, how would I want it then? Maybe I would want them to change the bed linen often. I hate having cold feet; I would want them to warm the wheat pillow. Those small things.

Lisa: Because your daughter might not know that.

Mark: No

Lisa: You may have a very good relationship with your children, but they may not know everything.

Mark: You don’t think to say it [you have to write it down].

Strategies for safeguarding the partnership involved transforming personal knowledge about the older person into shared organisational knowledge. The strategies included:

- Developing and implementing structured action plans based on older persons’ life stories and expressed needs.
- Ensuring that staff have the professional competence, empathy and language skills required for accurate documentation and effective implementation of care plans.
- Clarifying professional roles and responsibilities.

## Discussion

By focusing on organisational opportunities for person-centred care in residential aged care facilities from the perspectives of managers and staff, this study contributes empirical knowledge on resources and strategies that may support the sustained implementation of person-centred care in these settings. The findings show that managers and staff perceived person-centred care as dependent on organisational foundations that enabled staff to create a safe and supportive environment, maintain continuity of care and respond to older persons’ desires and needs. When interpreted through the GPCC framework,^3^ these foundations could be understood as supporting concrete strategies for initiating, working and safeguarding partnerships with older persons. Thus, the deductive analysis is not to be considered a separate inductive finding, but as a way of understanding how organisational foundations may be translated into person-centred care in practice.

The findings are consistent with previous research showing that leadership, organisational culture, staff education, continuity, communication and documentation are important for implementing person-centred care.^36–38^ The contribution of the present study is therefore not the identification of entirely new organisational conditions, but a contextualised understanding of how these conditions are perceived to work together in residential aged care. Person-centred care was described as requiring more than individual commitment or professional competence. It depended on organisational arrangements that allowed staff to know older persons over time, interpret verbal and non-verbal expressions, adapt routines, document preferences and follow-up agreed actions in everyday care.

The findings also emphasise the need for a shared understanding of person-centred care among managers and staff. Previous research has shown that person-centred care is associated with economic benefits for healthcare organisations^39–41^ and improved work environments.^27 37^ In Sweden, person-centred care is further reinforced through legal requirements under the Patient Act.^42^ Despite these incentives, organisational and structural challenges, such as limited staff continuity, regulatory constraints, time constraints and documentation demands, continue to impede implementation.^5 18^ The present findings add that leadership may function as a practical buffer between organisational demands and everyday care by clarifying responsibilities, supporting prioritisation and trusting staff’s professional judgement. This is in line with Moore *et al*’s^5^ findings that supportive leadership and staff education could be facilitators for implementation of person-centred care.^5^ However, organisational opportunities and barriers operate at several levels, including person, team and organisational levels^43^ indicating a need for leadership education that integrates both ethical foundations and practical application.^44^

Consistent with previous research, the findings underscore the importance of continuity in staffing and care provision.^36 37 45^ Low staff turnover has been associated with higher care quality, as well as improved staff well-being and job satisfaction.^36 37 46^ The present study supports these findings and illustrates why continuity matters for person-centred care. Continuity was not only described as a staffing issue, but as a relational condition that enabled staff to develop familiarity with older persons, recognise subtle changes, understand preferences, adapt communication and know when to act or step back. Rather than focusing solely on recruitment, the results indicate a need for managerial strategies aimed at retaining skilled and experienced staff. Although increasing workforce size may raise staffing costs as described by Spilsbury *et al*^36^, improved care quality may reduce costs related to adverse events, administrative burden, turnover, sick leave and the use of temporary staff.^36^ In relation to the present findings, this indicates the importance of long-term workforce planning, ongoing professional development and supportive leadership in achieving person-centred care.

The findings on responsiveness in everyday care are consistent with research emphasising the importance of the person’s narrative, preferences and lived experience in person-centred care.^4^ However, the present study shows that responsiveness is not only an interpersonal skill. It also requires organisational flexibility, including time to listen, opportunities to return later, routines that can be adapted and support for involving relatives when needed. Without such conditions, responsiveness risks depending on individual staff members’ personal commitment rather than being embedded in ordinary practice.

The deductive analysis provided practical insights into how organisational foundations may be translated into daily practice. By applying the GPCC framework,^3^ the findings complement existing research^5 47 48^ by illustrating how supportive environments, continuity and responsiveness may contribute to initiating, working and safeguarding partnerships with older persons. However, the GPCC framework^3^ should be understood as an interpretive lens for the deductive analysis, rather than as the source of the primary empirical findings. The inductive analysis identified what managers and staff perceived as organisationally important, while the deductive analysis provided one way of understanding how these conditions may support person-centred routines in practice.

Consistent documentation of discussions and agreed action plans involving older persons, relatives and staff appeared essential for sustaining partnerships, as supported by previous research.^49 50^ The present findings support this but also suggest that documentation alone is insufficient. To support person-centred care, documentation must be actively used in communication, follow-up and shared responsibility. Otherwise, there is a risk that documented preferences remain disconnected from daily care practices. Organisational support is therefore crucial in enabling managers and staff to create, maintain and follow-up opportunities for person-centred care.

### Strengths and limitations

A key strength of this study is the inclusion of participants from multiple professional groups working across different residential aged care facilities, providing a broad range of professional and organisational perspectives. Previous literature suggests that engaging relevant target groups enhances the likelihood of generating meaningful and representative data.^33 51–53^ The focus group methodology enabled interaction and collective reflection, which are central to developing shared understandings of experiences and practices.^34^ In this study, the discussions were rich and generated sufficient data to answer the study aim.

The analysis considered both dominant patterns and less prevalent perspectives, in line with focus group methodology, which emphasises attention to frequency, intensity and the context in which views are expressed. Consequently, both commonly shared views and more nuanced or less frequently expressed perspectives were included in the analysis. Divergent perspectives were considered important for understanding the complexity of organisational opportunities for person-centred care and were therefore not treated as outliers to be excluded.

The deductive use of the GPCC framework^3^ helped interpret how the organisational foundations identified in the inductive analysis could support person-centred routines in practice. At the same time, the framework shaped the interpretation and should not be understood as having generated the primary findings. The inductive analysis identified what managers and staff perceived as organisationally important, whereas the deductive analysis provided a way of understanding how these conditions may contribute to initiating, working and safeguarding partnerships.

Some limitations should also be acknowledged. The focus groups consisted of between three and seven participants. While some authors^33 51–53^ argue that the ideal number of participants is between four and eight, smaller groups can yield valuable insights when interaction is strong. Other scholars emphasise that group dynamics are more important than the number of participants.^34^ In this study, the discussions were lively, and participants openly shared both positive and negative experiences, supporting the credibility of the findings. In assessing sample adequacy, we drew on the concept of information power,^54^ which proposes that the more relevant information a sample holds for the study aim, the fewer participants are required. Information power is influenced by sample specificity, study aim, use of established theory, quality of dialogue and analytical strategy. High sample specificity, a clearly delimited aim, theoretical grounding, strong dialogue and an in-depth analytical approach reduce the need for a large sample.^54^ As this study employed focus groups, principles from Krueger and Casey^32^ and Kitzinger^33^ were also considered. Focus groups generate data through participant interaction, and rich, engaged dialogue can therefore increase information power. The participants in this study, comprising managers and staff working in residential aged care, were well suited to the aim of exploring organisational opportunities for person-centred care. They shared relevant organisational experience while representing sufficient variation in professional roles and organisational responsibilities to stimulate discussion. This aligns with both sample specificity according to Malterud *et al*^54^ and methodological guidance for focus groups.^32 33^

Participants did not provide feedback on the study findings, as member checking is not generally considered consistent with focus group methodology. Rather, the interactive group discussion itself is regarded as an important source of validation through participants’ shared exploration and clarification of perspectives during the focus group.^32 33^

One focus group was conducted online at the participants’ request, whereas the remaining groups were conducted in person. Although this may be viewed as a limitation, Schneider *et al*^55^ describe advantages of online focus groups, including geographical flexibility.^55^ Conducting one session online enabled the participation of healthcare managers who might otherwise have been unable to participate. The quality and depth of discussion in this group were comparable to those of the in-person sessions.

A further limitation is that the study reflects the perspectives of managers and staff and does not include the views of older persons living in residential aged care facilities or their relatives. This is particularly important in a study on person-centred care, as the absence of older persons’ own voices may introduce a professional bias. The findings therefore primarily show how organisational opportunities for person-centred care are understood by those responsible for organising and providing care. They cannot determine whether older persons experience these organisational conditions as person-centred in everyday life. This limitation should, however, be understood in relation to the broader research project of which this study forms part. The larger project explores person-centred approaches to residential aged care from multiple perspectives, including older persons, healthcare and social service staff and managers. The present study contributes specifically with professional and organisational perspectives. Future research that includes older persons’ and relatives’ perspectives is needed to examine whether and how the organisational foundations identified here correspond to lived experiences of person-centred care.

## Conclusion

This study contributes a contextualised understanding of organisational conditions that managers and staff perceived as important for enabling person-centred care in residential aged care. The findings suggest that person-centred care depends on organisational foundations that support safe and supportive environments, continuity of care and responsiveness to older persons’ preferences and needs. Rather than relying solely on individual staff commitment, person-centred care requires workforce stability, sustained managerial support, clear roles and responsibilities and organisational routines that enable staff to recognise, document and respond to older persons’ needs. When interpreted through the GPCC framework^3^, these organisational foundations can be understood as supporting strategies for initiating, working and safeguarding partnerships in everyday care. Despite strong policy endorsement of person-centred care, implementation remains challenging and requires long-term organisational commitment. Future research should evaluate organisational and system-level strategies that support workforce stability, leadership, continuity, communication and structured action planning in residential aged care. Research should also include older persons’ and relatives’ perspectives to examine whether the organisational conditions identified in this study are experienced as person-centred in everyday life.

## Supplementary material

10.1136/bmjopen-2026-118924 online supplemental file 1

## Data Availability

Data are available upon reasonable request.
