# Supplementary material for: Organisational opportunities for person-centred care in residential aged care: a focus group study with managers and staff
Source: BMJ Open. 2026 Jul 31;16(7):e118924. doi: 10.1136/bmjopen-2026-118924 (PMC13435985; doi:10.1136/bmjopen-2026-118924)
Supplement: Supplementary file 2 [file bmjopen-2026-118924.reviewer_comments.pdf]

PEER REVIEW HISTORY

BMJ Open publishes all reviews undertaken for accepted manuscripts. Reviewers are asked to complete a checklist review form and are provided with free text boxes to elaborate on their assessment. These free text comments are reproduced below.

ARTICLE DETAILS

Title (Provisional)

Organisational opportunities for person-centred care in residential aged care: A focus group study with managers and staff

Authors

Østby, Roar Hermansen; Dahlin-Ivanoff, Synneve; Najar, Jenna; Lood, Qarin

---

VERSION 1 - REVIEW

**Reviewer**	**1**
**Name**	**Buret, Laetitia**
**Affiliation**	**Liege University, General Practice Department - Primary Care and Health Research Unit**
**Date**	**23-Apr-2026**
**COI**	**None**

-Summary of the study and the authors

This qualitative study explores the organisational opportunities for implementing person-centred care in residential care homes for older people in Sweden, from the perspective of managers and professionals.The authors employ a focus group methodology (n=23 participants, 5 groups) involving a range of professions (nurses, care assistants, therapists, managers). The analysis combines an inductive thematic approach followed by a deductive interpretation based on the GPCC (Gothenburg Person-Centred Care) framework.The results identify opportunities at two levels, organisational foundations (safe environment, continuity, responsiveness) and practical implementation via partnership strategies.The study concludes that the sustainable implementation of person-centred care depends heavily on organisational factors and managerial commitment.

-About the authors:

The authors are from a Swedish university, and some of them, including the first author, are affiliated with the Centre for Person-Centred Care. Furthermore, their use of the GPCC framework and its Swedish contextualisation suggest credible academic expertise in the field of person-centred care.

-Novelty and contribution

The study makes a modest but relevant contribution.

It provides a concrete operationalisation of the concept of person-centred care at an organisational level.

It focuses on organisational conditions, which have been studied less extensively than individual interventions. However, the results confirm the existing literature (continuity, leadership, organisational culture, training) and offer little new insight. The use of the GPCC framework, whilst relevant, strongly influences the interpretation rather than generating new findings.

This study is therefore primarily of interest as an exploration of elements useful for implementing the concept of person-centred care in the specific context of care homes for the elderly in Sweden.

-Regarding the methodology

The use of focus groups is appropriate for exploring organisational dynamics and collective norms.

The sample is relevant and diverse. The questions in the interview guide are aligned with the research objective.The analysis process is rigorous and the dual inductive and deductive approach is interesting.

Major point:

1/ A major limitation of this study on person-centred care is the absence of the patients' perspective. I would recommend that the authors explicitly address this gap as a key bias. Based on my research, could this study form part of a broader study that includes patients? If so, this should at least be mentioned.

2/ As currently written, the article suggests that it provides relevant but not novel insights compared to what is found in the international literature. The article would gain in credibility by explaining even more explicitly what new insights the study offers.

3/ The analysis is primarily descriptive; it would gain in depth by going beyond the descriptive aspects and offering an analysis of the mechanisms. For example, regarding continuity, why is it difficult, how does it influence the professional-patient relationship, and what conditions make it possible or not?

Minor point

1/ Some sections (particularly the introduction and the results) contain long sentences or ones that are sometimes difficult to follow. A linguistic review would improve the overall readability.

2/ Terms related to person-centred care ("person-centred care", "partnership") sometimes appear to be used interchangeably.

3/ Adding a few more recent references (if available) would strengthen the contemporary relevance

+ Minor typographical corrections

Reviewer **2**

Name **Silies, Katharina**

Affiliation **University of Lübeck, Institute for Social Medicine and Epidemiology, Nursing Research Unit**

Date **18-May-2026**

COI **None**

Title: Organisational opportunities for person-centred care in residential aged care: A focus group study with managers and staff

I have been very pleased to have the opportunity to review your manuscript.

You present results from a focus group study on the implementation of person-centred care in long-term care, focusing on organisational conditions to foster implementation of this concept. Participants of the focus groups were of different professions relevant to person-centred care in long-term care. You present results of an inductive and a deductive analysis strand. Overall, your manuscript is well written and interesting to read, and targets an important topic.

Still, there are some areas for improvement. Your reporting of recruitment strategies and methods of analysis needs some more details. In your results, the two parts of your analysis, inductively and deductively developed themes, are not yet well connected into a holistic picture. Some consistency is lacking between summaries in the abstract, main results and discussion / conclusion section. I am sure, these concerns can be remedied, please find my detailed comments below.

Detailed review:

Major comments (content)

Abstract

1. P3,L22: Participants – registered nurses are missing here.

2. Conclusions: I recommend to move the first sentence of the conclusions to the results section. The remainder suffices for the conclusion.

3. However, I think results and conclusions in the abstract do currently not match with or summarise well what is reported in the main part of the manuscript under these sections.

4. P4,L29: Strengths and limitations: formed homogeneous yet varied focus groups … - this is in itself not well understandable, perhaps you could instead mention that they were in same professions groups but from different facilities and areas? Or so I understand this point.

5. P4, L33: One focus group … - I think this is a minor limitation and not necessary to mention in this section.

Introduction/Background

6. I have no comments, it is well explained and logically leads up to the objective and research question.

Methods

7. Study setting: I appreciate that you explain a bit about the setting as this is actually very different internationally.

8. Recruitment of participants: I recommend to give some more information on the recruitment. How many facilities were contacted (to which the 28 invited persons belonged)? Who decided, which facilities or professionals to approach? Were there personal contacts or how were they chosen?

9. Data analysis: P11, L24: were written down … - in which way? Paper and pencil or did you use some form of software for the analysis?

10. P11, L31: Following the inductive analysis … - I am not sure how this is to be understood: were inductively developed themes analysed deductively or were original data analysed a second time with the deductive approach?

11. You provide a reporting checklist, which is appropriate and appreciated. For several items you report "N/A". I recommend to pick these items up in your report and if you did not perform these steps, state so in the main manuscript and refer to the page in the checklist. E.g. Item 25: Why did you not provide a coding tree? Why is it not applicable? Did you not create one? Item 27, Software: if you did not use any software, this should be mentioned in the main text.

12. Patient and public involvement: please explain in which way the reference group with representatives was involved in the design of the study.

Results

13. Figure 1: In this figure, the results of the two methods of analysis, inductive and deductive are separated, it does not show, how themes are interrelated or built upon each other. I find it difficult to understand the connection between the inductive and the deductive analysis.

14. You provide quotes from three of four focus groups. What about the content of the assisting nurses focus group? In general: it is unclear to me, in which way the different focus

groups contributed to the different themes. Were the perspectives of the professions differing or were there similarities?

15. I miss some more information about the participants, for example: did they know each other? From how many facilities did they come? What were the characteristics of their facilities?

16. P15, L15: healthcare managers: could you explain whether healthcare managers are responsible for one or for more than one facility?

17. P15, L40: Ava – I do not quite understand what she is talking about, I would appreciate some contextual information in the surrounding text.

18. P18, L10: Turning foundations … - I don't understand how the deductively developed themes are connected to the inductively developed themes or whether they are connected at all.

19. You present interview data to underline and illustrate your inductively developed themes. Could you also provide quotes for the deductively analysed themes? This can be done in a supplement if the word count does not permit integration into the main manuscript.

Discussion

20. In the summary at the beginning of the discussion, you highlight the central role of partnerships as a key finding – why do you not relate to the inductive themes you developed, or to the overarching two parts of your results? I do not see partnership as prominent result presented in the main section.

Conclusion

21. P24, L15: The results suggest … - I think more specific implications for research and practice can be derived and formulated from your results. Currently, you remain a bit on an abstract level.

Minor comments (language, tables and figures, …)

22. P21, L13: In Sweden, person-centred is further … - is there a word missing? Care?

Good luck with the revision!

VERSION 1 - AUTHOR RESPONSE

Reviewer: 1

We thank Reviewer 1 for the constructive comments regarding the contribution, analytical depth, and the absence of older persons' perspectives. These comments helped us revise the manuscript to present the study's contribution more cautiously and clearly.

Major point:
1/ A major limitation of this study on person-centred care is the absence of the patients' perspective. I would recommend that the authors explicitly address this gap as a key bias. Based on my research, could this study form part of a broader study that includes patients? If so, this should at least be mentioned.

Response: We agree that the absence of older persons' perspectives is an important limitation and potential source of bias. We have now explicitly addressed this in the Background (pages 5-6), the Strengths and limitations (page 29), and the Conclusion (page 30). We have also clarified that the present study forms part of a broader research project exploring person-centred approaches to residential aged care from multiple perspectives, including older persons living in residential aged care facilities, healthcare and social service staff, and managers. Please see Background (pages 5-6) and the Strengths and limitations (page 29).

Because the study setting is residential aged care, we use the term "older persons" rather than "patients" throughout the manuscript.

2/ As currently written, the article suggests that it provides relevant but not novel insights compared to what is found in the international literature. The article would gain in credibility by explaining even more explicitly what new insights the study offers.

Response: Thank you for this important observation. We agree that the contribution should be presented more cautiously. We have revised the Abstract, Results, Discussion, and Conclusion to avoid overstating novelty. The revised manuscript now clarifies that the study does not identify entirely new organisational conditions, but contributes a contextualised understanding of how commonly recognised conditions, such as leadership, continuity, communication, documentation, competence, and organisational flexibility, are perceived to work together in residential aged care. We have also clarified that the GPCC framework was used as a deductive interpretive lens after the inductive analysis, rather than as the source of the primary empirical findings.

3/ The analysis is primarily descriptive; it would gain in depth by going beyond the descriptive aspects and offering an analysis of the mechanisms. For example, regarding continuity, why is it difficult, how does it influence the professional-patient relationship, and what conditions make it possible or not?

Response: We agree. We have deepened the analysis and revised the findings significantly. In particular, we have expanded the sections on continuity, managerial support, responsiveness, documentation, and action plans. We now explain how continuity influences relationships between staff and older persons by enabling familiarity, trust, interpretation of subtle changes, and responsiveness to preferences and needs. We also clarify how continuity may be constrained by staff turnover, temporary staff, insufficient information transfer, and limited organisational support.

Minor point

1/ Some sections (particularly the introduction and the results) contain long sentences or ones that are sometimes difficult to follow. A linguistic review would improve the overall readability.

Response: We have revised the manuscript throughout to improve readability. Long sentences have been shortened, repetitive wording has been reduced, and transitions between sections have been clarified.

2/ Terms related to person-centred care ("person-centred care", "partnership") sometimes appear to be used interchangeably.

Response: Thank you. We have revised the manuscript to distinguish more clearly between person-centred care as the broader approach and partnership as one component or routine within the GPCC framework. We now clarify that partnership was not identified as a separate inductive theme but was used in the deductive interpretation to understand how organisational foundations may support person-centred routines.

3/ Adding a few more recent references (if available) would strengthen the contemporary relevance

Response: We have added the following references:
Pot A, Rabheru K, Chew M. Person-centred long-term care for older persons: a new Lancet Commission. The Lancet (British edition). 2023;401(10390):1754-5.

Churruca K, Falkland E, Saba M, Ellis L, Braithwaite J. An integrative review of research evaluating organisational culture in residential aged care facilities. BMC health services research. 2023;23(1):857-11.

Backman A, Ahnlund P, Lövheim H, Edvardsson D. Nursing home managers' descriptions of multi-level barriers to leading person-centred care: A content analysis. International journal of older people nursing. 2024;19(1):e12581-n/a.

Lood Q, Carlsson Lalloo E, Bergholtz J, Barenfeld E. Leadership education to support person-centred health and social care: A scoping review of empirical literature. PloS one. 2026;21(6):e0350991.

Shen K, McGarry B, Gandhi A. Health care staff turnover and quality of care at nursing homes. JAMA internal medicine. 2023;183(11):1247-54.

+ Minor typographical corrections

Response: We have corrected typographical errors throughout the manuscript.

Reviewer: 2

We thank Reviewer 2 for the detailed and constructive review. We have revised the manuscript to clarify the recruitment process, analytical procedure, connection between the inductive and deductive findings, consistency across Abstract, Results, Discussion, and Conclusion, and participant/contextual information.

Detailed review:

Major comments (content)

Abstract

1. P3,L22: Participants – registered nurses are missing here.

Response: Thank you for noting this. Registered nurses have now been added to the Participants section of the Abstract.

2. Conclusions: I recommend to move the first sentence of the conclusions to the results section. The remainder suffices for the conclusion.

Response: We have revised the Abstract so that the Results section summarises the generated themes and main findings, while the Conclusions section focuses on implications.

3. However, I think results and conclusions in the abstract do currently not match with or summarise well what is reported in the main part of the manuscript under these sections.

Response: We have substantially revised the Abstract to better reflect the structure and content of the Results and Conclusions in the abstract. The Results section now reports the two connected themes and the three subthemes. The Conclusions now focuses on what the findings imply for person-centred care in residential aged care.

4. P4,L29: Strengths and limitations: formed homogeneous yet varied focus groups … - this is in itself not well understandable, perhaps you could instead mention that they were in same professions groups but from different facilities and areas? Or so I understand this point.

Response: We have revised this bullet point to clarify that the focus groups were organised to provide diverse perspectives on organisational conditions for person-centred care, see page 3.

5. P4, L33: One focus group … - I think this is a minor limitation and not necessary to mention in this section.

Response: We agree and have removed this point.

Introduction/Background

6. I have no comments, it is well explained and logically leads up to the objective and research question.

Response: Thank you. We have retained the overall structure of the Introduction/Background while making minor revisions to address Reviewer 1's comment about the absence of older persons' perspectives and to clarify that the study is part of a broader research project.

Methods

7. Study setting: I appreciate that you explain a bit about the setting as this is actually very different internationally.

Response: Thank you. We have retained and clarified the description of the Swedish residential aged care setting.

8. Recruitment of participants: I recommend to give some more information on the recruitment. How many facilities were contacted (to which the 28 invited persons belonged)? Who decided, which facilities or professionals to approach? Were there personal contacts or how were they chosen?

Response: We have revised the Participants section (pages 7-9) significantly to provide more detail about recruitment. We now clarify how facilities were approached, who contacted managers, how eligible participants were identified, how many facilities and persons were involved, and how participants were invited.

9. Data analysis: P11, L24: were written down … - in which way? Paper and pencil or did you use some form of software for the analysis?

Response: We have clarified how codes, preliminary themes, and analytical notes were documented during analysis. See page 11.

10. P11, L31: Following the inductive analysis … - I am not sure how this is to be understood: were inductively developed themes analysed deductively or were original data analysed a second time with the deductive approach?

Response:

Thank you. We have clarified the relationship between the inductive and deductive stages of analysis. The inductive analysis was conducted first and generated the empirical theme and subthemes. Thereafter, the codes, extracts, and inductively developed findings were re-examined in relation to the GPCC framework. Thus, the deductive stage was not a separate

analysis that generated independent empirical findings, but an interpretive step used to understand how the inductive findings could be related to the GPCC routines. Please see page 11.

11. You provide a reporting checklist, which is appropriate and appreciated. For several items you report "N/A". I recommend to pick these items up in your report and if you did not perform these steps, state so in the main manuscript and refer to the page in the checklist. E.g. Item 25: Why did you not provide a coding tree? Why is it not applicable? Did you not create one? Item 27, Software: if you did not use any software, this should be mentioned in the main text.

Response: We have revised the Analysis section on page 11 to state that no formal coding tree was used. We have also clarified that the analysis was conducted in word.

12. Patient and public involvement: please explain in which way the reference group with representatives was involved in the design of the study.

Response: We have expanded the Patient and public involvement section to describe how the reference group contributed to the design of the study, page 12.

Results

13. Figure 1: In this figure, the results of the two methods of analysis, inductive and deductive are separated, it does not show, how themes are interrelated or built upon each other. I find it difficult to understand the connection between the inductive and the deductive analysis.

Response: Thank you for this important and constructive comment. We appreciate that the current version of the figure may give the impression that the inductive and deductive analyses are presented as separate and unconnected processes.
To address this lack of clarity, we have revised the figure to more explicitly illustrate the relationship between the two analytic approaches.

14. You provide quotes from three of four focus groups. What about the content of the assisting nurses focus group? In general: it is unclear to me, in which way the different focus groups contributed to the different themes. Were the perspectives of the professions differing or were there similarities?

Response: Thank you for this thoughtful and important comment. We appreciate the opportunity to clarify both the role of the assistant nurses' focus group and how the different focus groups contributed to the analysis.
First, regarding the assistant nurses' focus group, their data were included in the analytic process and contributed to the development of the themes. The reason fewer quotations

from this group were presented in the text was not due to exclusion, but rather a matter of selection for illustrative purposes. We have now revised the manuscript to include quotations from the assistant nurses to better reflect their contribution.

Second, we acknowledge that the current presentation may give the impression that the contributions of the different focus groups are not clearly articulated. However, it is important to clarify that our aim was not to compare professional perspectives across groups. Instead, the focus groups were used to capture a breadth of experiences related to the phenomenon under study, and the analysis was conducted across the dataset as a whole. While there were both similarities and nuances in how participants expressed their experiences, these variations were not analysed in terms of professional differences, as this was beyond the scope and aim of the study.

15. I miss some more information about the participants, for example: did they know each other? From how many facilities did they come? What were the characteristics of their facilities?

Response: We agree that additional information about the participants and their context strengthens the transparency of the study.
We have now clarified this in the Methods section (page 9). The facilities varied in terms of size and organisational structure. Only public ownership were represented.

Regarding familiarity, all participants were acquainted with each other through their work within the same facility. This composition was considered appropriate, as it allowed for both shared experiences and diversity of perspectives within the focus group discussions.
These details have now been added to the manuscript to improve clarity (page 9).

16. P15, L15: healthcare managers: could you explain whether healthcare managers are responsible for one or for more than one facility?

Response: Thank you for this important clarification. In our study context, healthcare managers were responsible for more than one residential aged care facility. Unfortunately, specific data regarding the exact number of facilities per manager were not systematically collected and are therefore not available.

17. P15, L40: Ava – I do not quite understand what she is talking about, I would appreciate some contextual information in the surrounding text.

Response: We have revised the surrounding text to provide context for this quote. We now explain that the quote concerns uncertainty about responsibilities for basic equipment and assistive devices, and how managerial support helped clarify responsibilities so that staff could focus on older persons' needs. Please see page 15.

18. P18, L10: Turning foundations … - I don't understand how the deductively developed

themes are connected to the inductively developed themes or whether they are connected at all.

Response: We have revised the Methods, Results, Discussion and Conclusion sections to clarify this connection. The inductive analysis generated the main empirical findings concerning organisational foundations. The deductive analysis then interpreted these foundations in relation to the GPCC framework. We have also revised Figure 1 to show this relationship more clearly.

19. You present interview data to underline and illustrate your inductively developed themes. Could you also provide quotes for the deductively analysed themes? This can be done in a supplement if the word count does not permit integration into the main manuscript.

Response: Thank you for this thoughtful suggestion. We appreciate the value of including illustrative quotes to enhance transparency. We have added quotes for the deductively analysed themes, see pages 17-19, and 22.

Discussion

20. In the summary at the beginning of the discussion, you highlight the central role of partnerships as a key finding – why do you not relate to the inductive themes you developed, or to the overarching two parts of your results? I do not see partnership as prominent result presented in the main section.

Response: We agree and have revised the Discussion. The Discussion now reflects the two-part structure of the Results. It first summarises the inductively developed organisational foundations and then explains how these were interpreted through the GPCC framework. We no longer present partnership as the central empirical finding.

Conclusion

21. P24, L15: The results suggest … - I think more specific implications for research and practice can be derived and formulated from your results. Currently, you remain a bit on an abstract level.

Response: We have significantly revised the Conclusion to include more specific implications for practice and future research. The revised Conclusion now highlights the need for organisational strategies that support continuity, managerial support, documentation, communication, and responsiveness, and calls for future studies that include older persons and relatives. See page 29-30.

Minor comments (language, tables and figures, …)

22. P21, L13: In Sweden, person-centred is further … - is there a word missing? Care?

Response: Thank you. We have corrected this sentence on page 24.

In addition to the changes made in response to specific comments, we have revised the manuscript throughout to improve clarity, readability, and consistency. We have reduced repetition between the Results and Discussion, clarified the terminology related to person-centred care and partnership, corrected typographical errors, and ensured that the Abstract, Results, Discussion, and Conclusion are aligned.

We again thank the Editor and Reviewers for their constructive feedback, which has helped us improve the clarity, transparency, and contribution of the manuscript.

VERSION 2 - REVIEW

Reviewer 2

Name Silies, Katharina

Affiliation University of Lübeck, Institute for Social Medicine and Epidemiology, Nursing Research Unit

Date 11-Jul-2026

COI

It has been a pleasure to review your revised manuscript. You addressed all comments appropriately, well done. I especially appreciate your clarifications and further explanations in the methods section.

One minor comment: please reference the software of the “word document” at first appearance.

Good luck with your further process!
